# Humoral immune escape by current SARS-CoV-2 variants BA.2.86 and JN.1, December 2023

**DOI:** 10.2807/1560-7917.ES.2024.29.2.2300740

**Published:** 2024-01-11

**Authors:** Lara M Jeworowski, Barbara Mühlemann, Felix Walper, Marie L Schmidt, Jenny Jansen, Andi Krumbholz, Etienne Simon-Lorière, Terry C Jones, Victor M Corman, Christian Drosten

**Affiliations:** 1 Institute of Virology, Charité - Universitätsmedizin Berlin, corporate member of Freie Universität Berlin, Humboldt-Universität zu Berlin and Berlin Institute of Health, Berlin, Germany; 2 German Centre for Infection Research (DZIF), partner site Charité, Berlin, Germany; 3 Institute for Infection Medicine, Christian-Albrechts-Universität zu Kiel and University Medical Center Schleswig-Holstein, Campus Kiel, Kiel, Germany; 4 Laboratory Dr. Krause und Kollegen MVZ GmbH, Kiel, Germany; 5 G5 Evolutionary Genomics of RNA Viruses, Institut Pasteur, Université Paris Cité, Paris, France; 6 National Reference Center for Viruses of Respiratory Infections, Institut Pasteur, Paris, France; 7 Centre for Pathogen Evolution, Department of Zoology, University of Cambridge, Cambridge, United Kingdom; 8 Labor Berlin - Charité Vivantes GmbH, Berlin, Germany; * These authors contributed equally to the work and share the first authorship.; ** These authors contributed equally to the work and share the last authorship.

**Keywords:** COVID-19, Immunity, SARS-CoV-2, Variant JN.1

## Abstract

Variant BA.2.86 and its descendant, JN.1, of SARS-CoV-2 are rising in incidence across Europe and globally. We isolated recent JN.1, BA.2.86, EG.5, XBB.1.5 and earlier variants. We tested live virus neutralisation of sera taken in September 2023 from vaccinated and exposed healthy persons (n = 39). We found clear neutralisation escape against recent variants but no specific pronounced escape for BA.2.86 or JN.1. Neutralisation escape corresponds to recent variant predominance but may not be causative of the recent upsurge in JN.1 incidence.

Since first emergence in late 2021, variants of the severe acute respiratory syndrome coronavirus 2 (SARS-CoV-2) Omicron lineage (Phylogenetic Assignment of Named Global Outbreak (Pango) lineage designation B.1.1.529) with changing immune escape properties have continued to cause waves of incidence in humans on a global scale [1]. During 2023, the BA.2.86 lineage with an unusually large number of additional mutations in the spike protein has been a cause of concern. Whereas serum neutralisation escape was not found to be increased over previously circulating strains [2-13], the derived JN.1 sublineage with an additional substitution in the spike protein (L455S) currently shows stronger increase in circulation than BA.2.86 worldwide [14,15].

## Serum neutralisation of BA.2.86 and JN.1 compared with earlier variants

To assess whether the increased spread of JN.1 compared with BA.2.86 is related to its ability to escape pre-existing immunity, we assessed neutralisation titres in 39 sera against seven different variants: B.1, BA.2, BA.5, XBB.1.5, EG.5.1, BA.2.86 and JN.1. The sera stem from 39 individuals affiliated with our institution, representing a predominately young (median age 36 years, range 26–60 years; 16 males, 23 females) and healthy population resident in Berlin and surrounding areas (Table). Detailed data on sampled individuals can be seen in Supplementary Table S1. All individuals had received at least a primary immunisation series against severe acute respiratory syndrome coronavirus 2 (SARS-CoV-2) using mRNA (Comirnaty, Pfizer, New York, the United States/BioNTech, Mainz, Germany or Spikevax, Moderna Biotech, Madrid, Spain) or vector-based (Vaxzevria, AstraZeneca, Cambridge, the United Kingdom) vaccines and 32 reported at least one infectious episode confirmed by PCR (n = 31) or antigen test (n = 1). All reported infections occurred during the period when the Omicron variant circulated.

**Table:** Demographic and clinical characteristics of persons with serum samples for neutralisation of severe acute respiratory syndrome coronavirus 2 antibodies, Germany, September 2023 (n = 39)

Characteristics	Total (range)	Non-XBB exposure (range)	XBB exposure^a^ (range)
Number of participants	39	30	9
Median age (years)	36 (26–60)	36 (26–60)	38 (29–60)
**Sex**			
Female	23	20	3
Male	16	10	6
**Number of vaccinations**			
3	23	19	4
4	16	11	5
**Median number of days past last vaccination**	642 (245–705)	644.5 (245–705)	380 (297–652)
3 vaccinations total	649 (598–705)	650 (616–705)	644 (598–652)
4 vaccinations total	325 (245–425)	323 (245–425)	334 (297–380)
**Number of infections**			
0	7	7	0
1	19	16	3
2	13	7	6
**Median number of days past last infection**	306 (26–612)	355 (198–612)	121 (26–220)
1 infection total	355 (29–612)	408 (270–612)	148 (29–190)
2 infections total	209 (26–427)	283 (198–427)	89 (26–220)

^a^ Omicron XBB exposure was assigned according to available sequencing data or due to predominance of Omicron XBB at the time of infection (March 2023 onwards) or high reactivity against XBB determined by plaque reduction neutralisation tests.

Sera were taken in September 2023 (8–29 September), at a time when the SARS-CoV-2 EG.5.1 variant had dominated circulation in Germany and the Berlin region for at least 1.5 months. A graph on the circulating variants in Germany is depicted in Supplementary Figure S1. Neutralisation titres were determined by plaque reduction neutralisation tests (PRNT) performed on Vero E6 (African green monkey kidney epithelial) cells expressing the transmembrane serine protease TMPRSS 2 (National Institute for Biological Standardization and Control product 100978). Neutralisation titres can be seen in Supplementary Table S1. Titres were determined as the dilution where 50% of plaques were neutralised. Geometric mean titres (GMT) and fold changes were estimated using the ‘titretools’ package [16] in R [17].

**Figure 1:**
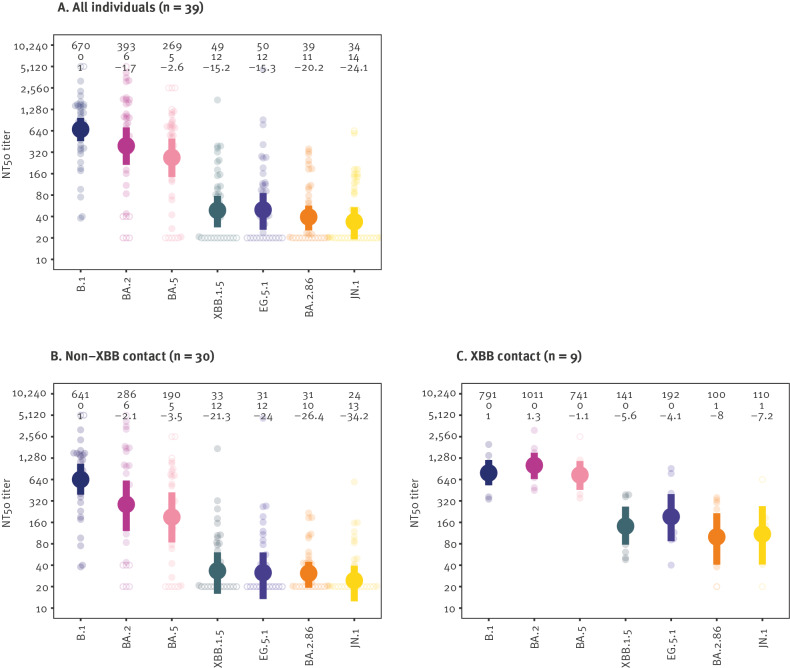
Serum neutralisation titres against severe acute respiratory syndrome coronavirus 2 variants BA.1, BA.2, BA.5, XBB.1.5., EG.5.1, BA.2.86 and JN.1, Germany, September 2023 (n = 39)

We found highest titres against variant B.1, followed by BA.2 and BA.5, consistent with the presence of immunity from vaccination. Compared with the ancestral B.1 variant, there was a similar reduction in titres for XBB.1.5 and EG.5.1 (15.2-fold 95% highest posterior density interval (HPDI): 10.4–23.4) and 15.3-fold (95% HPDI: 9.6–29.5) reduction), with 12 individuals showing no measurable neutralising reactivity against either variant (Figure 1). The variants are described in Supplementary Table S2. Titres against BA.2.86 were further reduced (20.2-fold; 95% HPDI: 13.1–32.4)), with 11 of 39 individuals having no detectable titres. Compared with BA.2.86, the JN.1 variant did not show any additional reduction in titre (1.1-fold (95% HPDI: 1.6 to -1.3) reduction). When splitting the cohort into individuals exposed to an XBB variant (n = 9) or not exposed (n = 30), we also found limited evidence for additional escape of JN.1 compared with BA.2.86 (Figure 1).

Sera were titrated by plaque reduction neutralisation tests (PRNT) against B.1, BA.2, BA.5, XBB.1.5, EG.5.1, BA.2.86 and JN.1.

Pale circles correspond to individual serum titres and empty circles correspond to sera with non-detectable titres. Larger circles and confidence intervals correspond to the geometric mean titre and 95% highest posterior density intervals of each variant. Geometric mean titres, number of sera without detectable neutralisation and fold drop relative to B.1 are given (vertically) at the top of each variant in all panels.

## Synopsis with findings from other studies

**Figure 2:**
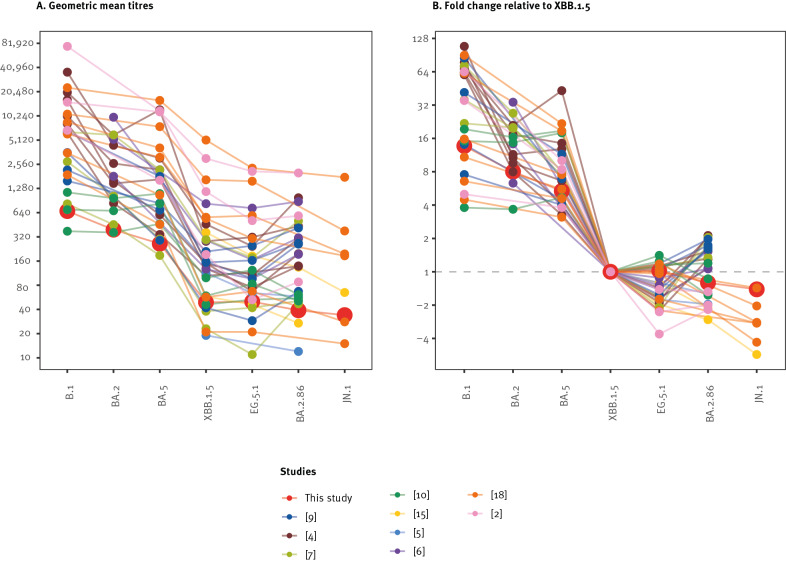
Comparison of neutralisation titres to severe acute respiratory syndrome coronavirus 2 variants, Germany, September 2023 (n = 39) with recent studies assessing escape of BA.2.86 and JN.1

Few published studies have assessed neutralisation of BA.2.86 using live virus neutralisation assays [2,9-12], and none so far have done so for JN.1. We compared the geometric mean titres and fold change measured in different studies, including those using pseudoviruses, that presented titrations of BA.2.86 and/or JN.1 [2-7,9,10,14,15,18]. The studies titrated between two and six groups of sera with different vaccination and infection histories. We included 10 studies with 33 groups of sera in formal evaluation (exclusions were because of incomplete variant coverage). Overall, our live virus assays generally yielded lower geometric mean titres and lower fold change values (Figure 2). Our findings of limited additional escape of JN.1 are in contrast to two studies [15,18] that used vesicular stomatitis virus (VSV) pseudotypes and cohorts with a higher proportion of individuals with an infection or vaccination history with XBB variants.

Panel A: Geometric mean titres for 33 groups of sera from the 10 studies mentioned in the legend.

Panel B: Fold change relative to XBB.1.5 was calculated from geometric mean titres as shown in panel A. Fold change compared with XBB.1.5 was calculated from the extracted geometric mean titres using the log2diff function in the ‘titretools’ package (version 4.2.0 [16],) in R (version 4.2.0 [17]), as described [24]. The data presented in this manuscript are highlighted by larger symbols and a darker grey line.

## Discussion

Since the first circulation of the Omicron BA.1 variant (Phylogenetic Assignment of Named Global Outbreak (Pango) lineage designation B.1.1.529) in late 2021, different Omicron variants have spread successively [1]. In 2023, circulation was largely dominated by variants descendent from the XBB sublineage that arose from recombination of two Omicron BA.2 variants (BJ.1 and BM.1.1.1), in particular variants XBB.1.5, XBB.1.9 and XBB.1.16 [19]. In summer 2023, different XBB variants began to convergently acquire substitutions at positions 356, 403, 453, 455, 456, 478 or 486 [2,19]. For example, the EG.5.1 variant descended from XBB.1.9, but additionally acquired three substitutions in the spike protein (Q52H, F456L, F486P). No difference in growth or pathogenicity in hamsters was found between EG.5.1 and XBB.1.5 [20,21], but EG.5.1 showed more escape from neutralisation than XBB.1.5 [2,3,21]. In August 2023, the BA.2.86 variant was first detected in Israel and Denmark and designated by the World Health Organization (WHO) as a variant under monitoring on 17 August 2023 and subsequently a variant of interest on 21 November 2023 (https://www.who.int/docs/default-source/coronaviruse/21112023_ba.2.86_ire.pdf). The BA.2.86 variant descends from BA.2, but has acquired 43 additional substitutions, including 34 in the spike protein. The proportion of BA.2.86 sequences has been increasing globally in October 2023 (https://www.who.int/docs/default-source/coronaviruse/21112023_ba.2.86_ire.pdf), and BA.2.86 has been found to have a higher effective reproduction number compared with EG.5.1 [22]. Despite the additional substitutions in BA.2.86, multiple studies have reported its neutralisation escape as similar to that of circulating XBB variants such as XBB.1.5 and EG.5.1 [2-13]. Furthermore, BA.2.86 shows equal or poorer growth in cell culture systems than EG.5.1 [2,12,22] and is less pathogenic in the hamster model compared with EG.5.1, BA.2 or BA.2.75 [22,23]. However, BA.2.86 has increased angiotensin-converting enzyme 2 (ACE2) binding affinity compared with XBB.1.5 and EG.5 [2,3,6], raising concern that it may be able to tolerate additional substitutions in the spike protein that negatively affect ACE2 binding affinity, allowing it to escape neutralisation more strongly than the original BA.2.86 variants. The JN.1 variant is such a BA.2.86 descendant variant and was designated as a separate variant of interest on 18 December 2023 (https://www.who.int/docs/default-source/coronaviruse/18122023_jn.1_ire_clean.pdf). This variant (JN.1) has an additional substitution in the spike protein (L455S) and currently shows stronger increase in circulation than BA.2.86 worldwide [14,15].

The present study provides an initial assessment of neutralisation escape for the current JN.1 variant that is rapidly increasing in incidence in many countries. About one third of our cohort of relatively young and healthy subjects had low or no detectable neutralisation against the most recently circulating variants XBB.1.5, EG.5.1, BA.2.86 and JN.1. This suggests that a considerable fraction of the population may be susceptible to reinfections during the coming winter months in the northern hemisphere. Limitations of our study include the rather small group of individuals, their comparatively young age, as well as our reliance on only one format of virus neutralisation assay that may limit the comparability with other studies. However, we found good agreement between studies overall.

## Conclusion

Based on the present data and other studies, it seems unlikely that neutralisation escape is the facilitating principle behind the present increase in JN.1 incidence as opposed to earlier strains. If so, we would have expected strong reductions in neutralisation activity, such as the decrease between BA.5 and XBB.1.5 that is deemed responsible for the upsurge of cases over winter 2022/23 in North America. Changes other than neutralisation escape may affect viral fitness and deserve further study.

## Supplementary Data

296000 Supplementary Material
