# Author’s correction for Euro Surveill. 2024;29(2)

**DOI:** 10.2807/1560-7917.ES.2024.29.3.240118c1

**Published:** 2024-01-18

**Authors:** Eurosurveillance editorial team

**Affiliations:** 1 European Centre for Disease Prevention and Control (ECDC), Stockholm, Sweden

In the article entitled ‘Humoral immune escape by current SARS-CoV-2 variants BA.2.86 and JN.1, December 2023’ by Jeworowski et al. published on 11 January 2024, in the title of Figure 1, the name of a severe acute respiratory syndrome coronavirus 2 (SARS-CoV-2) variant was incorrectly reported as BA.1. The correct variant is B.1, and this was rectified at the request of the authors on 12 January 2024.

