# Supplementary material for: Humoral immune escape by current SARS-CoV-2 variants BA.2.86 and JN.1, December 2023
Source: Euro Surveill. 2024 Jan 11;29(2):2300740. doi: 10.2807/1560-7917.ES.2024.29.2.2300740 (PMC10785204; doi:10.2807/1560-7917.ES.2024.29.2.2300740)
Supplement: Supplementary Material [file 23-00740_DROSTEN_Supplement.pdf]

Supplementary information

This supplementary material is hosted by Eurosurveillance as supporting information alongside the article 'Humoral immune escape by current SARS-CoV-2 variants BA.2.86 and JN.1', on behalf of the authors, who remain responsible for the accuracy and appropriateness of the content. The same standards for ethics, copyright, attributions and permissions as for the article apply. Supplements are not edited by Eurosurveillance and the journal is not responsible for the maintenance of any links or email addresses provided therein.

Supplementary figures and tables

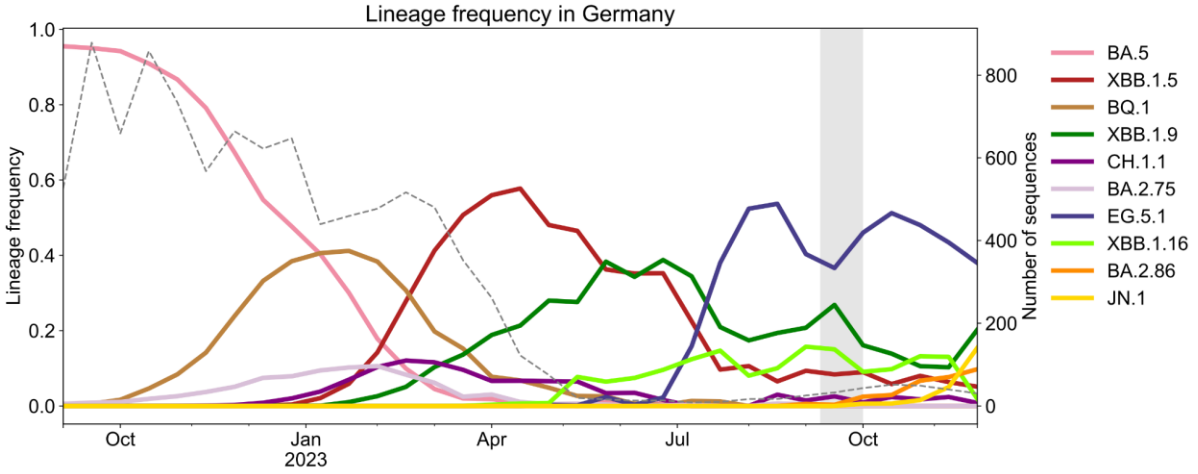

Figure S1: Circulating variants in Germany up to 2023-12-15. Sequences from Germany with >= 95% genome coverage sampled after 2022-06-30 were downloaded from GISAID on 2023-11-24 (EPI_SET_231218xq, doi: 10.55876/gis8.231218xq). Clades were assigned using nextclade [1] with default options. Sequences with unclear sampling dates were omitted. Clade '21L (BA.2)' was manually constrained to not include any BA.2.86 sequences or their descendants, based on the lineage assignment given by GISAID. Within the BA.2.86 sequences, JN.1 sequences were identified based on their Pangolin lineage. Lineage frequency was plotted on a biweekly basis using pandas (version 1.3.5) [2] in Python (version 3.7.8). The lines show the frequency of particular lineages, as indicated by the color. The gray dashed line shows the total number of sequences. Lineage frequencies and total number of sequences are shown aggregated over two weeks. The time interval where blood collection took place (2023-09-08 to 2023-09-29) is marked in gray

Table S1: Sample characteristics and neutralization titers. Inf: infection, vac: vaccination.

sample ID	sample characteristics							NT50 titers						
	**age (years)**	**sex**	**no. of vac**	**days past last vac**	**no. of inf**	**days past last inf**	**XBB contact**	**B.1**	**BA.2**	**BA.5**	**XBB.1.5**	**EG.5.1**	**BA.2.86**	**JN.1**
**50503**	30-39	f	4	323	1	569	no	3190.39	1869.14	1072.11	97.08	89.47	44.1	20.28
**50564**	40-49	f	4	327	0	0	no	1202.09	1028.2	750.3	106.81	56.33	185.04	38.81
**50565**	40-49	f	3	664	1	601	no	523.06	236.62	68.5	<20	<20	<20	<20
**50566**	30-39	f	3	648	2	344	no	565.29	254.85	132.08	<20	<20	<20	25.92
**50568**	30-39	f	3	644	2	427	no	176.01	179.88	124	<20	<20	20.31	21.9
**50570**	30-39	f	3	645	1	355	no	1412.25	1004.27	518.02	44.5	29.64	104.57	30.02
**50571**	40-49	m	4	322	1	415	no	1494.02	1581.75	898.3	64.21	54.51	61.22	20.35
**50574**	20-29	m	3	645	1	291	no	75.06	83.52	42.28	<20	23.87	40.66	20.2
**50575**	20-29	f	3	618	2	268	no	1507.68	1756.52	728.94	155.35	107.91	187.12	156.32
**50576**	30-39	f	4	245	1	475	no	>5120	>5120	>2560	104.63	77.8	96.73	49.63
**50577**	30-39	m	4	309	1	451	no	816.73	536.06	378.03	39.31	39.38	20.52	<20
**50580**	40-49	m	3	649	1	595	no	230.37	43.8	<20	<20	<20	<20	<20
**50582**	30-39	f	3	653	1	612	no	608.51	327.76	155.63	39.09	39.89	22.36	<20
**50583**	40-49	f	3	661	0	0	no	39.97	<20	<20	<20	<20	<20	<20
**50585**	30-39	f	4	425	1	320	no	1638.69	975.97	555.19	83.72	41.21	109.59	88.5
**50587**	40-49	m	3	672	1	480	no	1494.31	779.2	705.05	76.83	50.27	54.34	<20
**50595**	30-39	f	4	296	0	0	no	476.21	<40	27.04	<20	<20	23.52	<20
**50596**	40-49	f	3	662	0	0	no	37.86	<20	<20	<20	<20	<20	<20
**50598**	40-49	f	3	652	2	283	no	608.47	540.45	170.65	20.8	<20	30.18	48.07
**50601**	30-39	m	3	647	0	0	no	189.27	<40	<20	<20	<20	<20	<20
**50603**	30-39	f	4	394	1	346	no	304.14	<40	20.64	<20	<20	<20	<20
**50604**	20-29	f	4	318	2	198	no	1439.07	1783.15	1149.01	247.33	271.25	42.87	100.18
**50606**	60-69	f	3	660	1	322	no	>5120	3564.46	>2560	321.84	192.68	42.82	158.42

	sample characteristics							NT50 titers						
**50607**	30-39	m	3	642	1	401	no	856.26	390.11	319.34	31.79	31.25	<20	<20
**50612**	30-39	f	4	349	1	270	no	403.93	160.41	286.89	44.57	119.96	22.57	41.5
**50615**	40-49	m	3	616	1	276	no	1141.01	332.31	311.46	81.03	160.7	41.77	118.74
**50617**	40-49	m	4	349	0	0	no	362.58	109.38	77.46	<20	<20	<20	<20
**50641**	30-39	m	3	650	0	0	no	96.36	<20	<20	<20	<20	<20	<20
**50642**	30-39	f	3	662	2	209	no	1351.12	4175.54	>1280	1735.17	4638.37	216.74	591.57
**50647**	30-39	f	3	705	2	365	no	2302.84	3249.91	845.1	180.87	265.47	48.1	82.48
**50501**	30-39	m	4	344	2	28	yes	1393.78	3138.78	>2560	365.28	777.55	244.48	185.08
**50502**	30-39	f	4	297	2	26	yes	338.87	1106.53	593.74	61.01	90.73	78.61	123.82
**50504**	20-29	m	3	644	1	148	yes	662.01	680.37	348.84	77.86	94.2	37.75	40.97
**50578**	20-29	m	4	314	1	190	yes	832.54	450.4	397.16	47.77	40.1	65.79	50.21
**50581**	60-69	m	4	380	1	29	yes	990.1	959.93	691.53	149.63	115.35	44.38	<20
**50590**	40-49	f	3	643	2	220	yes	364.21	1752.22	734.06	390.65	409.13	356.25	144.61
**50591**	30-39	f	3	598	2	56	yes	1986.8	1525.05	1204.52	390.65	280.76	302.71	182.94
**50599**	30-39	m	3	652	2	121	yes	588.23	491.62	788.15	234.6	912.16	328.77	>640
**50614**	30-39	m	4	334	2	196	yes	1108.47	822.5	548.33	50.89	112.45	<20	145

Table S2: Description of isolates.

Variant	Spike substitutions relative to Wuhan-Hu-1 (EPI_ISL_402125)	Stock-ID	Additional substitutions and minor variants >5% of reads in S and >20% of reads in rest of the genome	GISAID accession number
B.1	D614G	V146_984_V.V.p2	Synonymous minor variant C24616T (S), 10% of reads	EPI_ISL_18474149
BA.2	T19I; L24S; P25-A27del; G142D; V213G; G339D; S371F; S373P; S375F; T376A; D405N; R408S; K417N; N440K; S477N; T478K; E484A; Q493R; Q498R; N501Y; Y505H; D614G; H655Y; N679K; P681H; N764K; D796Y; Q954H; N969K	V166_27758_V.V.p1	Non-synonymous minor variant ORF1a:L3829F, 8% of reads	EPI_ISL_18516395
BA.5	T19I; L24S; P25-A27del; H69-V70del; G142D; V213G; G339D; S371F; S373P; S375F; T376A; D405N; R408S; K417N; N440K; L452R; S477N; T478K; E484A; F486V; Q498R; N501Y; Y505H; D614G; H655Y; N679K; P681H; N764K; D796Y; Q954H; N969K	V150_28261_A.V.p1	Non-synonymous substitution ORF1a:L3249V	EPI_ISL_18474151
XBB.1.5	T19I; L24S; P25-A27del; V83A; G142D; Y144del; H146Q; Q183E; V213E; G252V; G339H; R346T; L368I; S371F; S373P; S375F; T376A; D405N; R408S; K417N; N440K; V445P; G446S; N460K; S477N; T478K; E484A; F486P; F490S; Q498R; N501Y; Y505H; D614G; H655Y; N679K; P681H; N764K; D796Y; Q954H; N969K	V145_41742_V.V.p3	Non-synonymous minor variant ORF1a:F3829L, 48% of reads, ORF1a:Q4289R, 22% of reads, Synonymous minor variant C9940T, 46% of reads, T22672C, 35% of reads	EPI_ISL_18370592
EG.5.1	T19I; L24S; P25-A27del; Q52H; V83A; G142D;	V140_43769_V.V.p1	Non-synonymous minor variant,	EPI_ISL_18370587

	Y144del; H146Q; Q183E; V213E; G252V; G339H; R346T; L368I; S371F; S373P; S375F; T376A; D405N; R408S; K417N; N440K; V445P; G446S; F456L; N460K; S477N; T478K; E484A; F486P; F490S; Q498R; N501Y; Y505H; D614G; H655Y; N679K; P681H; N764K; D796Y; Q954H; N969K		S:D839E, 5% of reads	
BA.2.86	ins17MPLF; T19I; R21T; L24S; P25-A27del; S50L; H69-V70del; V127F; G142D; Y144del; F157S; R158G; N211I; L212del; V213G; L216F; H245N; A264D; I332V; G339H; K356T; S371F; S373P; S375F; T376A; R403K; D405N; R408S; K417N; N440K; V445H; G446S; N450D; L452W; N460K; S477N; T478K; N481K; V483del; E484K; F486P; Q498R; N501Y; Y505H; E554K; A570V; D614G; P621S; H655Y; N679K; P681R; N764K; D796Y; S939F; Q954H; N969K; P1143L	V139_44057_u.V.p3	Non-synonymous minor variant ORF1a:T2495I, 21% of reads	EPI_ISL_18221650
JN.1	ins17MPLF; T19I; R21T; L24S; P25-A27del; S50L; H69-V70del; V127F; G142D; Y144del; F157S; R158G; N211I; L212-; V213G; L216F; H245N; A264D; I332V; G339H; K356T; S371F; S373P; S375F; T376A; R403K; D405N; R408S; K417N; N440K; V445H; G446S; N450D; L452W; L455S; N460K; S477N; T478K; N481K; V483del; E484K; F486P; Q498R; N501Y; Y505H; E554K; A570V; D614G; P621S; H655Y; N679K; P681R; N764K; D796Y; S939F; Q954H; N969K; P1143L	V148_44042_V.V.p1	None	EPI_ISL_18474148

References

1. Aksamentov I, Roemer C, Hodcroft E, Neher R. Nextclade: clade assignment, mutation calling and quality control for viral genomes. J Open Source Softw. 2021;6(67):3773.

2. Pandas documentation — pandas 2.1.3 documentation. Available from: https://pandas.pydata.org/docs/index.html.
